# Early Speech and Language Development in Children With Nonsyndromic Cleft Lip and/or Palate: A Meta-Analysis

**DOI:** 10.1044/2019_JSLHR-19-00162

**Published:** 2019-12-13

**Authors:** Hope Sparks Lancaster, Kari M. Lien, Jason C. Chow, Jennifer R. Frey, Nancy J. Scherer, Ann P. Kaiser

**Affiliations:** a Program of Speech and Hearing Science, College of Health Solutions, Arizona State University, Tempe; b Department of Counseling and Special Education, Virginia Commonwealth University, Richmond; c Department of Special Education & Disability Studies, The George Washington University, Washington, DC; d Department of Special Education, Peabody College of Vanderbilt University, Nashville, TN

## Abstract

**Objective:**

The aim of the study was to conduct a meta-analysis of research examining the early speech and language functioning of young children, birth to age 8;11 (years;months), with nonsyndromic cleft lip and/or palate (NSCL/P) compared to their peers without NSCL/P.

**Method:**

We conducted a random-effects metaregression using 241 effect sizes from 31 studies comparing 955 young children with NSCL/P to 938 typically developing peers on measures of speech and language functioning. Moderators were sample characteristics (i.e., age, cleft type, publication year, and study location) and measurement characteristics (i.e., speech sample material, language modality and domain, and assessment type).

**Results:**

Young children with NSCL/P scored significantly lower on measures of speech and language compared to children without NSCL/P. Children with NSCL/P had smaller consonant inventories (standardized mean difference effect size [ES_g_] = −1.24), less accurate articulation (ES_g_ = −1.13), and more speech errors (ES_g_ = 0.93) than their peers. Additionally, children with NSCL/P had poorer expressive (ES_g_ = −0.57) and receptive (ES_g_ = −0.59) language skills than their peers. Age and assessment type moderated effect sizes for expressive language. As children with NSCL/P aged, their expressive language performance became more similar to their peers. Expressive language effect sizes from parent reports and observational language measures (estimated effect size = −0.74) were significantly lower than those from standardized norm-referenced tests (estimated effect size = −0.45).

**Conclusions:**

These findings suggest that young children with NSCL/P experience delays relative to their peers across multiple speech and language constructs. Differences between children with NSCL/P and their typically developing peers appear to decrease with age.

**Supplemental Material:**

https://doi.org/10.23641/asha.11356904

Nonsyndromic cleft lip and/or palate (NSCL/P) affects the development of speech mechanism, and children with NSCL/P are at risk for speech and language delays (Chapman, 2011; Scherer, Williams, & Proctor-Williams, 2008). The speech and language skills of children with NSCL/P, however, have not been systematically studied across the early childhood developmental period (birth to 8 years of age). In addition, the existing developmental literature has reported mixed findings about the speech and language characteristics of young children with NSCL/P, the degree to which their development differs from same-age peers, and whether differences are maintained over time (i.e., as children age). Furthermore, these inconsistent or inconclusive data have constrained the identification of evidence-based, assessment, and intervention best practice guidelines for this population of children who are at high risk for language and/or speech delays. Given the important role of speech and language skills in both academic and social emotional growth, it is important to better understand the existing literature and use these findings as a basis for future research and clinical practice.

## Summary of Early Development and Delays

Some children with NSCL/P present with speech concerns persisting into the toddler, preschool, and early school–age years. These concerns include smaller consonant inventories following palate repair compared to their typically developing peers (Jones, Chapman, & Hardin-Jones, 2003), reduced speech accuracy compared to peers at age 3 years (Klintö et al., 2014), as well as reduced speech accuracy relative to their peers during early school–age years (Lohmander & Persson, 2008). Young children with NSCL/P may also demonstrate greater use of atypical phonological processes during the preschool years compared to their peers (Chapman, 1993), which may further delay their speech development. In addition, children with NSCL/P often use compensatory articulation errors more frequently than their peers (Klintö et al., 2014); such errors may develop in the context of velopharyngeal dysfunction and become habituated speech error patterns. Recently, data from an intercenter speech outcomes project conducted in North America indicated that 5-year-old children with NSCL/P were less articulate (i.e., had poorer speech articulation skills) compared to their same-age peers (Chapman et al., 2017).

Expanding upon these early differences in speech development, there is also a documented relationship between early speech articulation skills of children with NSCL/P and the diversity of their expressive vocabulary development in early childhood. More specifically, articulation delays are often associated with reduced expressive vocabulary of children with NSCL/P (Chapman, Hardin-Jones, & Halter, 2003). Delayed expressive vocabulary acquisition may be attributed, in part, to young children with NSCL/P continuing to produce fewer words that contain high-pressure phonemes (e.g., stop phonemes) because they were unable to produce these sounds prior to palate repair (Hardin-Jones & Chapman, 2014). Regardless, given vocabulary upon school entry is one of the greatest predictors of later academic success (Hoff, 2013), early delays in speech development, smaller vocabularies (Scherer, Boyce, & Martin, 2013; Young, Purcell, & Ballard, 2010), and delayed language acquisition (Cavalheiro, Lamônica, de Vasconsellos Hage, & Maximino, 2019; Hardin-Jones & Chapman, 2014) place children with NSCL/P at higher risk for persistent language delays (Morris & Ozanne, 2003; Priester & Goorhuis-Brouwer, 2008) and at greater risk for challenges in school. In fact, it has been reported that children with NSCL/P have higher incidence rates of language disorders compared to children without NSCL/P (Morgan et al., 2017; Richman, 1980; Watkins et al., 2018), which may result from speech deficits (Chapman et al., 2003), cognitive differences (Roberts, Mathias, & Wheaton, 2012), or hearing loss (Schönweiler et al., 1999).

While some researchers report relatively lower language skills of children with NSCL/P compared to their typically developing peers, other researchers have reported no significant differences between young children with NSCL/P and their peers by age 5 years, suggesting that, as young children with NSCL/P mature, they “catch up” to their peers (Boyce, Kilpatrick, Reilly, Da Costa, & Morgan, 2018; Collett, Leroux, & Speltz, 2010). These mixed findings and limited longitudinal data motivated the current meta-analysis comparing early language development of children with NSCL/P with their peers.

## Factors Affecting Speech and Language Development

The mixed findings reported in the research literature could be the result of differences across research samples (e.g., sample age, sample cleft repair timing) or differences in methods for assessing speech and language skills. Therefore, it is important not only to characterize potential differences in speech and language development but also to determine the roles of possible moderators of development (e.g., sample characteristics and assessment strategies).

Past research indicates a number of possible factors may influence speech and language development in children with NSCL/P. These factors include (a) sample characteristics (e.g., chronological age, cleft type, hearing status, surgical repair timing and type), (b) assessment approaches and measurement tools used to evaluate speech and language skills (e.g., speech sampling material, language domain and modality assessed, type of assessment), and (c) environmental conditions (e.g., socioeconomic status). Any number of factors could explain the variabilities in reported research findings about speech and language development in children with NSCL/P. However, not all of factors of interest are consistently reported in the literature, which limits possible systematic analyses of these factors.

Two descriptors consistently associated with speech and language development are chronological age and cleft type. As children with NSCL/P get older, the assumption is that their speech and language skills will improve and will eventually be within typical performance ranges (i.e., speech and language skills will be similar to same-age peers without NSCL/P). This assumption is not fully supported by research evidence (Chapman et al., 2017; Collett et al., 2010). We do not know how speech outcomes in children with NSCL/P are moderated by age; therefore, it is possible inconsistent findings across the research literature might be related to the age of participants. In the area of language development, research has reported that 5-year-old children with NSCL/P did not differ from their peers on measures of language functioning (e.g., receptive vocabulary, expressive vocabulary, verbal fluency; Collett et al., 2010); however, in their meta-analysis of cognitive abilities across the life span, Roberts et al. (2012) found that general language functioning of individuals with NSCL/P was below that of age-matched peers without NSCL/P through the life span.

A second factor related to individual differences is cleft palate type. Previous research found that the type of cleft (i.e., cleft lip only, cleft palate only, unilateral cleft lip and/or palate, bilateral cleft lip and/or palate) may influence speech articulation skills (Ha, Koh, Moon, Jung, & Oh, 2015; Lohmander-Agerskov, Söderpalm, Friede, Persson, & Lilja, 1994). For example, cleft type has been found to moderate outcomes within the NSCL/P population; individuals with cleft palate only had lower language skills compared to individuals with cleft lip and palate (Roberts et al., 2012). Current practices for comparing speech outcomes assume that speech outcomes differ according to cleft type (Long et al., 2011); however, some research (Choa et al., 2014) has indicated that the type of cleft does not influence speech outcomes, and there have been no research syntheses examining the potential moderation of speech and language outcomes by cleft type. The inconsistent findings regarding the impact of cleft type on speech and language outcomes warrant further investigation of cleft type as a moderator of speech and language skill development within children with NSCL/P.

Timing and type of surgical repair may be additional factors affecting speech and language development. The common assumption is that the earlier the primary palatal repair takes place, the better the speech outcomes, because early palatal repair will provide children with more time with an intact palate. There are two ways to “stage” primary palatal repair: one-stage (i.e., a single surgery) or two-stage (i.e., two surgeries to close the palate) repair. Staging could affect speech outcomes, because two-stage surgeries reduce the amount of time that children have with an intact palate during the crucial speech and language learning development period. The current meta-analysis explores the effects of palatal repair timing and staging on the speech articulation outcomes reported across studies.

Additionally, assessment methods may influence the measurement of speech and language development. Information about the effects of different assessment approaches and measurement tools on speech and language outcomes is limited. Previous findings have demonstrated that speech outcomes are related to speech sampling material (e.g., single words vs. connected speech; Klintö, Salameh, Svensson, & Lohmander, 2011), but the degree to which reported speech outcomes may differ as a function of specific measure and speech sample materials has not been investigated across studies. Three measures are useful for characterizing children's speech skills: (a) size and composition of consonant inventory, which provides a measure of the capacity for sound production regardless of whether the sound was used correctly; (b) speech accuracy, which characterizes how close the child's production is to the adult model; and (c) speech error type, which describes use of developmental speech errors and/or cleft-related speech errors. Investigating which speech measures are most sensitive to identifying differences between children with NSCL/P and children without cleft is critical given the importance of early speech articulation skills as indicators of adequate velopharyngeal function and as the foundation for early expressive vocabulary development.

For assessment of language development, it would be beneficial to know if language modality (i.e., expressive vs. receptive language), language domain (e.g., vocabulary vs. overall measure of language), and assessment type (e.g., standardized norm-referenced measures, naturalistic language sampling) affect the estimate of language skills. The selection of language measurement approaches may have contributed to inconsistent findings in past research. For example, it is often assumed that children with NSCL/Ps have typical receptive language skills, while their expressive language skills are delayed due to early delays in speech. This assumption should be evaluated in a meta-analysis across studies describing children with NSCL/P and their peers on measures of both expressive and receptive language skills.

Some researchers also have reported significant differences in vocabulary outcomes (Chapman et al., 2003), while other researchers have reported smaller differences on omnibus measures of language (i.e., measures assessing more than one aspect of language and reporting a single score; Morgan et al., 2017). Studies of vocabulary tend to agree that children with NSCL/P have smaller vocabularies and delayed vocabulary growth (Cavalheiro et al., 2019; Hardin-Jones & Chapman, 2014; Scherer, Boyce, et al., 2013; Scherer, Williams, et al., 2008; Young et al., 2010). In contrast, studies using omnibus language measures report more variable results, with some studies indicating language delays (Hardin-Jones & Chapman, 2014; Morgan et al., 2017; Morris & Ozanne, 2003; Watkins et al., 2018; Young et al., 2010) and others finding no difference from typically developing peers (Boyce et al., 2018; Collett et al., 2010; Priester & Goorhuis-Brouwer, 2008). Differences in findings between studies utilizing vocabulary measures and reporting scores from omnibus language measures make it difficult to determine the extent to which clefting affects only vocabulary development or all aspects of language development.

Finally, research studies that have relied exclusively on standardized test measures may have different findings than those including naturalistic language samples or parent report (Frey, Kaiser, & Scherer, 2018), as these assessment methods have different purposes and requirements and thus elicit different information from children. Therefore, it is important to consider how different assessment approaches may contribute to conclusions about differences in language development.

## Purpose

There are three clear gaps in our understanding of speech and language development in children with NSCL/P relative to their peers: (a) There are inconsistencies in past research examining development of early speech and language skills; (b) the examination of factors moderating speech and language outcomes has been limited, and when conducted, results have been variable across studies; and (c) there is no meta-analysis of studies comparing speech or language development in early childhood for children with NSCL/P to their typically developing peers. As a result of these gaps, the field lacks critical information necessary to inform research and clinical practice. The current meta-analysis addressed these gaps by examining the development of speech articulation and language functioning during early childhood (birth through 8 years of age). The analysis compared outcomes for children with NSCL/P to their typically developing peers and examined moderators of differences in outcomes. The research questions guiding this meta-analysis were as follows:

1. Do young children with NSCL/P differ from children without NSCL/P on three measures of speech articulation: consonant inventory, speech accuracy, and speech error type?
  a. Do sample characteristics (i.e., age, surgical timing and type, cleft type, publication year, and study location) and assessment approaches (i.e., speech sample material) moderate the difference between children with NSCL/P and their peers on measures of speech articulation skills?
2. Do young children with NSCL/P differ from children without NSCL/P on measures of expressive and receptive language?
  a. Do sample characteristics (i.e., age, cleft type, publication year, and study location) or assessment approaches (i.e., language domain, assessment type) moderate the difference between children with NSCL/P and their peers on measures of expressive or receptive language functioning?

## Method

### Search Strategy

We identified relevant studies through electronic searches of Academic Search Complete, CINAHL Plus With Full Text, ERIC, PsyArticles, PsycINFO, and MEDLINE. We used an integrated database search engine and limited the search to peer-reviewed articles, published in English, and included human participants. The search string entered into the search engine was ab(“cleft palate*”) NOT ab(“orthopedic*” OR “surgical technique*” OR “otitis media”). For this meta-analysis, there were no beginning date restrictions; all peer-reviewed reports through June 2018 were included. Unpublished manuscripts, reports, dissertations, and theses were excluded. This initial search yielded 3,965 search results. In addition to the electronic search, we reviewed the titles and abstracts from studies identified through a review of reference lists from relevant literature reviews and included primary studies. From this review of reference lists, we identified an additional 33 studies for potential inclusion.

### Inclusion and Exclusion Criteria

The procedures for this review were informed by the PRISMA (Preferred Reporting Items for Systematic Reviews and Meta-Analyses) guidelines (Moher, Liberati, Tetzlaff, Altman, & The PRISMA Group, 2009; see Supplemental Material S1 for the PRISMA checklist). To be included in the meta-analysis, studies were required to meet the following inclusion criteria: (a) empirical study; (b) group comparison design; (c) included at least one measure of articulation and/or language; (d) mean age of sample was between birth and 8 years 11 months or results desegregated by age and data for a sample within this age range were available; (e) reported sufficient data to calculate effect size or data were reported or provided upon request; (f) children with syndromic cleft were excluded from the sample or reported as a separate group from children with NSCL/P; (g) retrievable through journal, interlibrary loan, or database; and (h) published in English. We excluded studies that used (a) single-subject design or (b) a comparison sample that was another cleft group or clinical population.

### Screening Articles

**Figure 1.**
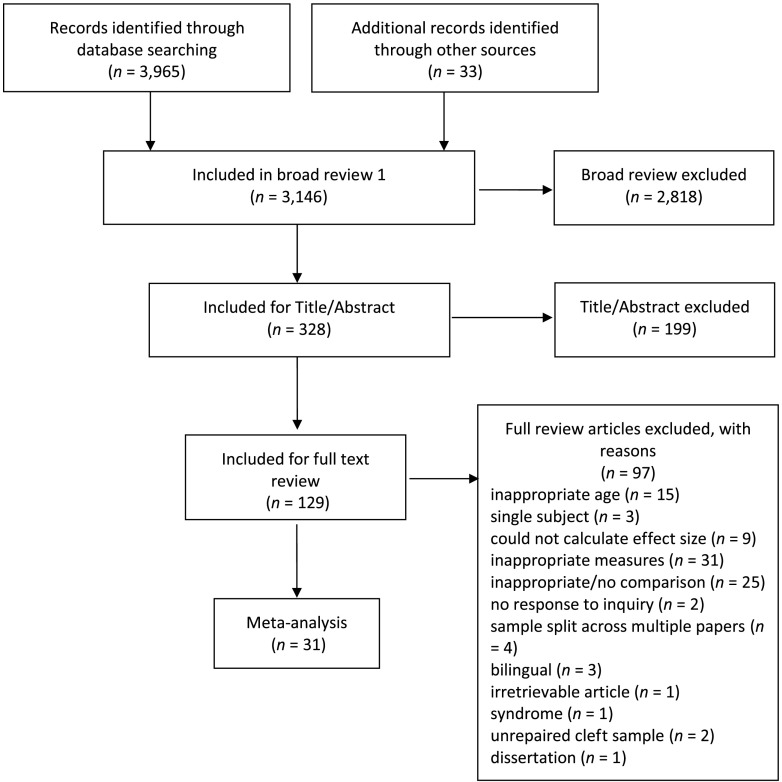
Flow chart of study identification and inclusion.

We conducted three rounds of screening. The flow chart for the search strategy is illustrated in Figure 1. In the first round, we excluded articles that did not meet study inclusion criteria (e.g., not an empirical study, not a group design, not about the relevant constructs) based on title and metatextual (e.g., language of publication, type of article) information. The second round used title and abstract information to exclude studies that did not meet study inclusion criteria. The third round of review used full-text information to determine final eligibility based on pre-established inclusion/exclusion criteria. After full-text screening, 31 articles were identified for inclusion in the meta-analysis. The first and second authors independently screened all articles in the third round. When there was a disagreement, the first and second authors discussed the study and came to consensus about study inclusion.

### Effect Size Selection and Coding

Results related to speech and language were analyzed separately to create cohesive data sets representing unique domains within the speech and language system. Operational definitions for the speech and language domains and examples of included measures are in Table 1. Within the speech domain, we created the following subconstructs based on measurement focus: (a) consonant inventory, (b) speech accuracy, and (c) speech error. For language, we created subconstructs based on language modality: (a) expressive and (b) receptive language. Ten of the included studies contributed effect sizes to both speech and language (Chapman, 2011; Chapman, Graham, Gooch, & Visconti, 1998; Chapman et al., 2003; Hardin-Jones & Chapman, 2014; Jones et al., 2003; Kummer, Lee, Stutz, Maroney, & Brandt, 2007; Priester & Goorhuis-Brouwer, 2008; Scherer & D'Antonio, 1995; Scherer, D'Antonio, & McGahey, 2008; Scherer, Williams, et al., 2008).

**Table 1.** Operational definitions and examples for all examined subconstructs by construct.

Subconstruct	Operational definition of subconstruct	Examples of included measures
Speech		
Consonant inventory	Examines the child's sound production without comparing the child's production to the correct form or the target production.	Consonant inventories, number of emerging consonants, number of final consonants
Speech accuracy	Compares the child's speech production to the target speech form, thus providing a metric of speech maturity.	Percentage of consonants correct, percentage of consonants correct by different manners of articulation (e.g., stops, fricatives)
Speech error usage	Provides information about the number of speech errors a child demonstrates.	Number of phonological processes, number of compensatory errors, number of cleft speech characteristics, raw score on a standardized speech assessment
Language		
Expressive language	Language skills involved in producing spoken language.	Naming pictures, mean length of utterances, parent report of words a child says, formulating sentences, story retell, number of different words
Receptive language	Language skills involved in understanding or comprehending spoken language. Measures do not require a child to produce a verbal response.	Receptive vocabulary as measured by selecting a picture, comprehending multiclausal sentences, composite scores from more than one receptive language subtests

Within the speech articulation domain, we excluded measures of speech intelligibility and speech acceptability because these constructs are based on listener perception and not on child performance. We did not analyze speech resonance outcomes (e.g., hypernasality) or other characteristics of velopharyngeal dysfunction (e.g., audible nasal emission). For language, we excluded measures that combined expressive and receptive language (e.g., the Preschool Language Scales total score [Zimmerman, Steiner, & Pond, 2012]) because this meta-analysis examined the effects of clefting on each subdomain.

### Moderator Selection and Coding

Meta-analytic procedures are ideal for examining possible confounding factors that influence past research findings. A limited number of these putative moderators consistently reported across studies were selected for this meta-analysis: mean chronological age, cleft type, speech sampling assessment protocol, language domain, palatal repair timing, palatal repair type, and assessment type (e.g., standardized direct assessment vs. parent report/observational sampling). Additionally, we coded each study for publication year and study location to determine if there were differences in research findings based on when or where a study was conducted. See Table 2 for the number of effect sizes per moderator.

**Table 2.** Number of effect sizes for each moderator.

Measurement characteristics	Speech	Language
Speech sampling context		
Single word	100	—
Connected speech	51	—
Palatal repair age	121	—
Palatal repair type		
One-stage repair	110	—
Two-stage repair	27	—
Language sampling context		
Vocabulary measure	—	59
Omnibus language measure	—	25
Measurement source		
Standardized test	—	61
Parent report or naturalistic observation measure	—	29

*Note.* Em dashes represent not applicable cells and are used for readability.

#### Sample Characteristics

Sample characteristics were coded for each effect size. These sample characteristics were mean age, cleft type, palatal repair timing, palatal repair type, publication year, and study location. Several articles reported multiple eligible cleft groups (e.g., multiple independent age groups, multiple cleft type groups, multiple surgical groups); therefore, effect sizes within a study might have different codes for mean age, cleft type, palatal repair timing, or palatal repair type. Publication year and study location did not vary within studies. Study location was coded as being conducted in the United States or other country. Mean age was calculated in months for each speech and language measure extracted. Cleft types were cleft palate only, unilateral cleft lip and palate, bilateral cleft lip and palate, cleft lip only, and a mixed cleft type group. For palatal repair timing, we extracted the mean age that palatal repair was completed for a sample. We coded palatal repair type as one-stage or two-stage repair. Publication year was coded as being published before or after 2006. This year was selected as the split in the sample, because in 2006, the Americleft Project was announced and officially funded in the United States. The Americleft Project encouraged researchers and clinicians to evaluate outcomes of care for children with NSCL/P.

#### Assessment Methods

Effect sizes were coded for speech sample material, language domain, and assessment type. For speech measures, the speech sample material was specified to indicate whether the speech measure was obtained from either a single word or connected speech context (e.g., sentence production, conversation sample). For language domain, measures were classified as vocabulary or omnibus language measure (i.e., tests that considered multiple types of language skills such as syntax, vocabulary, and semantics but yielded a single score). Assessment type was coded as standardized direct assessment (e.g., Clinical Evaluation of Language Fundamentals–Third Edition; Semel, Wiig, & Secord, 2003) or an observational or report measure (i.e., parent report or naturalistic language sample). All parent report measures, regardless of whether they were norm-referenced, were included in the observational or report measure category. For example, the MacArthur–Bates Communicative Development Inventories (Fenson et al., 2007) was considered an observational or report measure. This moderator was only coded for expressive language because none of the other domains had this dichotomy.

### Interrater Reliability Procedures

The first and second authors coded each article for effect sizes and moderators, and final coding decisions were made by consensus. Interrater reliability was conducted independently for speech and language. First, raters completed reliability training on seven articles for speech (44.67% of sample) and seven articles for language (35% of sample) until agreement was 100%. Then, interrater reliability was calculated on approximately 30% of each remaining sample (speech, *n* = 3; language, *n* = 3).

Single-measure intraclass correlations (ICCs) were calculated for effect size information (e.g., mean difference and sample size) and continuous moderators (e.g., mean age). Using Fleiss's (1999) guidelines, values of ICC above .75 were taken to represent excellent reliability. Kappa statistics were obtained for categorical moderators (e.g., cleft type, measure type, speech sample material, language domain). Using Landis and Koch's (1977) guidelines, kappa values of .61 were set as the minimum threshold for agreement, with interpretation for values as .61–.80 as acceptable agreement and .81–1 as perfect agreement.

### Analytical Procedures

In this meta-analysis, we applied robust variance estimation (RVE; Hedges, Tipton, & Johnson, 2010) to random-effects models to allow inclusion of multiple effect sizes from each study and to explore the impact of sample- and assessment-level moderators. Specifically, the effects of age, cleft type, measurement source, speech sample material, language domain, study location, and publication year were examined.

All analyses were conducted in R (v.3.3.2 “Sincere Pumpkin Patch;” R Core Team, 2017) using the RStudio interface. The R code and a list of R packages is provided in Supplemental Material S2. Data are available on the first author's ResearchGate profile. We used random-effects models for RVE main effect and metaregression models to account for within-study effect size dependency. Authors of reports with possibly shared samples were contacted to confirm that the samples were the same across articles. If the samples were shared across studies, the samples were treated like longitudinal studies, and effect sizes were selected from the oldest time point for each subconstruct. For studies that reported multiple cleft groups (e.g., cleft palate only, unilateral cleft palate and lip), an effect size was calculated for each cleft group and nested within the study identifier and the variable of interest. For each subconstruct, separate random effects analyses were conducted, and we ran an intercept-only model in which the estimate for the constant represented the average weighted effect size across studies (Tanner-Smith & Tipton, 2014).

#### Calculating Effect Sizes

All effect sizes were calculated by the first and second authors. Effect size values were calculated using the “Practical Meta-Analysis Effect Size Calculator” on the Campbell Collaboration website (Wilson, n.d.). The effect size was calculated using the reported cleft and comparison sample size, mean, and standard deviations, unless this information was unavailable, in which case, inferential test statistics (e.g., *t* test) were used. For four studies (Chapman, 2011; Chapman et al., 1998, 2003; Speltz et al., 2000), we used *t*-test results to calculate effect sizes, and for Hardin-Jones and Chapman (2014), we used *f*-test statistic results to calculate effect sizes. All effect sizes were transformed to bias-corrected Hedge's *g* (Lakens, 2013) using Formula 1:

$$d*1-\frac{3}{4*\left(n_{1}+n_{2}\right)-9}$$ (1)

#### Tests for Heterogeneity

The heterogeneity among effect sizes was examined using *Q*-statistics, *I* ^2^, and τ^2^ indices. The *Q*-statistic is a measure of between-studies variation considering within-study error. The *I* ^2^ index ranges from 0 to 100 and describes the proportion of total variance attributable to true between-studies heterogeneity. The standard *I* ^2^ benchmarks are low (25%), moderate (50%), and high (75%; Higgins, Thompson, Deeks, & Altman, 2003). τ^2^ is a second measure of between-studies variance and reflects the variance of the individual study effect sizes within the meta-analytic sample with smaller values indicating less difference between studies.

#### Moderator Analyses

A priori random-effects metaregression models including child, assessment, or study-level moderators were planned for adequately powered samples (i.e., inclusion of ≥ 10 effect sizes; Borenstein, Hedges, Higgins, & Rothstein, 2009) to investigate sources of variance in study effect sizes for each construct. We ran single-predictor RVE meta-regressions because, except for expressive language, models that included more than one predictor result in degrees of freedom < 4, despite application of small-sample size degrees of freedom correction procedure (which is the default for the “robumeta” package). We interpret the findings as exploratory and with caution because these models likely underestimate the true Type I error in the hypothesis tests (Tipton, 2015). Another caution about the single-predictor metaregressions is that results do not control for potential confounding effects of other moderators, as they are unaccounted for in the single-predictor model. Additionally, some single-predictor models remained underpowered (*df* < 4), which is possibly the result of imbalances in the data (Tipton, 2015).

#### Tests for Publication Bias

In meta-analysis, it is important to assess the likelihood that the publication process biased our findings (i.e., publication bias) and use the assessment of publication bias to contextualize meta-analytic findings. Following the recommendations of Chow (2018), we selected three methods to assess publication bias: funnel plots, Egger regression, and trim and fill. Funnel plots were graphed to visually examine potential publication bias. Standard errors for the funnel plots were calculated by taking the square root of the effect size variance obtained from the online effect size calculator. Egger's regression tests were also conducted to further examine small study effects. To ensure that the regressions would be adequately powered (i.e., inclusion of ≥ 10 effect sizes; Borenstein et al., 2009), Egger regressions were run within constructs as well. Additionally, we conducted trim-and-fill analyses to test for asymmetry in the data set based on the distribution of studies based on standard errors and effect size.

## Results

### Description of Studies

Descriptive statistics for the study and sample characteristics across all 31 studies and by construct are presented in Table 3. A total of 955 children with NSCL/P were compared with 938 children without NSCL/P. Studies dated from 1950 to 2018, with 45% published prior to 2006. Table 4 presents summary information about the samples of the included studies. Supplemental Material S3 contains the list of references with corresponding study identification number used for this study.

**Table 3.** Study and effect size characteristics for whole sample and by construct.

Coded variables	Total sample	Speech	Language
No. of articles	31	20	22
Study design			
Group	20	13	13
Longitudinal	10	6	8
Intervention with TD control	1	1	1
Reported reliability	19	16	12
Reported syndrome exclusion criteria	28	19	20
Sample size			
Total *N*	1,893	806	1,560
Total cleft *N*	955	405	786
Total TD *N*	938	401	774
Sample age, *M* (*SD*)			
Cleft	43.36 (22.44)	40.65 (20.74)	42.77 (22.65)
Noncleft TD peers	41.62 (22.93)	39.46 (21.28)	40.39 (22.83)
Percent male, *M* (*SD*)			
Whole sample	56.68 (9.53)	56.88 (10.79)	57.54 (8.30)
Cleft sample	55.91 (9.61)	56.24 (10.72)	57.37 (8.79)
Mean palatal repair age	11.44 (2.71)	11.26 (2.99)	11.83 (1.16)
Cleft type			
Included a CPO group	—	1	4
Included a UCLP group	—	7	8
Included a BCLP group	—	0	1
Mixed cleft group	—	14	15
Location			
USA	18	12	15
Other	13	8	7
Publication year			
< 2006	17	13	11
> 2006	14	7	11

*Note.* Em dashes represent not applicable cells and are used for readability. TD = noncleft typically developing peers; CPO = cleft palate only; UCLP = unilateral cleft lip and palate; BCLP = bilateral cleft lip and palate.

**Table 4.** Features of samples from each included study.

Citation	Study location	Cleft samples					Controls	
		*N*	*M*_age_	Age of palatal repair	Normal cognition	Normal hearing	*N*	*M*_age_
Broen et al. (1998)	USA	28	30	13.3	Y	Y	29	30
Chapman & Hardin (1992)	USA	10	26	25.5	Y	Y	5	25.2
Chapman et al. (1998)	USA	PreK: 10 School: 10	PreK: 48 School: 104	—	Y	Y	PreK: 10 School: 10	49
Chapman et al. (2003)	USA	15	21	12.47	Y	Y	15	21
Chapman (2011)	USA	28	67	12	Y	Y	28	68
Collett et al. (2010)	USA	CLP: 29 CPO: 28	84	—	U	U	53	84
Estrem & Broen (1989)	USA	5	22	14.3	Y	N	5	18.5
Fox et al. (1978)	USA	24	17.66	—	U	U	24	18.5
Hardin-Jones & Chapman (2014)	USA	37	27	12	Y	Y	22	—
Hentges et al. (2011)	England	93	84	—	U	U	77	84
Jocelyn et al. (1996)	Canada	26	24	—	Y	Y	16	24
Jones et al. (2003)	USA	14	16.43	12	Y	Y	14	16.43
Klintö et al. (2015)	Sweden	29	60	4.6	U	N	20	60
Klintö et al. (2011)	Sweden	20	60	—	U	U	20	60
Kummer et al. (2007)	USA	20	64	—	U	Y	47	65
Lee et al. (2009)	Taiwan	20	54	12	Y	Y	20	54
Lee et al. (2015)	Singapore	15	90	9	Y	Y	15	88
Lohmander & Persson (2008)	Sweden	20	84	6.55	U	Y	7	85
Luyten et al. (2014a)	Uganda	11	57	3.4	U	U	22	58
Luyten et al. (2014b)	Uganda and Belgium	Uganda: 12 Belgium: 12	Uganda: 58 Belgium: 56	Uganda: 3.3 Belgium: 11.1	U	U	Uganda: 12 Belgium: 12	49
Nakajima et al. (2001)	Japan	BCLP: 28 UCLP: 7 CPO: 33	54.19	13.3	Y	Y	52	—
Philips & Harrison (1969)	USA	67	49.19	—	U	Y	165	54.9
Priester & Goorhuis-Brouwer (2008)	The Netherlands	43	29	10	U	N	32	27
Scherer, D'Antonio, & McGahey (2008)	USA	10	27.4	12	Y	Y	10	20.2
Scherer et al. (2012)	USA	26	27.4	12	Y	Y	42	25.5
Scherer, Oravkinova, & McBee (2013)	USA and Slovakia	USA: 8 Slovakia: 8	24	11.1	Y	Y	USA: 8 Slovakia: 8	24
Scherer, Williams, & Proctor-Williams (2008)	USA	13	30	11.7	U	Y	13	30
Scherer & D'Antonio (1995)	USA	30	24.5	12.5	U	Y	30	23.8
Speltz et al. (2000)	USA	51	24	12.3	U	U	61	24
Snyder & Scherer (2004)	USA	25	30	1	U	Y	25	30
Willadsen & Enemark (2000)	Demark	33	13	7	Y	N	19	13

*Note.* Missing information is indicated by “—” character. Y = study indicated that children had to have normal cognition or normal hearing to be included in the cleft sample and either used medical records or completed testing of cognition/hearing themselves; PreK = preschool; CLP = cleft lip and palate; CPO = cleft palate only; U = study did not clarify if they used cognition or hearing as an inclusion criteria; *N* = study may have included children with low cognitive or hearing loss; BCLP = bilateral cleft lip and palate; UCLP = unilateral cleft lip and palate.

### Interrater Reliability

Interrater reliability was high across the board. Table 5 reports ICC and kappa statistics for speech and language effect sizes.

**Table 5.** Interrater reliability for effect size coding for speech and language data sets.

Effect size information	Intraclass correlation coefficients	
	Speech	Language
Cleft sample size	1	1
Compare sample size	1	1
Mean age of sample	1	1
Cleft mean	1	1
Compare mean	1	1
Cleft *SD*	.76	.97
	(*p* < .001)	(*p* < .001)
Compare *SD*	1	1
Effect size	1	1
Effect size variance	1	1
	**Kappa**	
	**Speech**	**Language**
Subconstruct coding	.94 (*p* < .001)	1 (*p <* .001)
Cleft type	1 (*p* < .001)	1 (*p* < .001)
Assessment type	—	.84 (*p* < .001)
Speech sample material	.77 (*p* < .001)	—
Language domain	—	1 (*p* < .001)

*Note.* Em dashes represent not applicable cells and are used for readability.

### Meta-Analytic Results by Subconstruct

For each subconstruct, average effect sizes represented the difference in the average number of standard deviation units between children with NSCL/P and children without NSCL/P. As expected, the random effects meta-analysis indicated that children with NSCL/P performed less well than children without NSCL/P on measures of consonant inventory, speech accuracy, expressive language, and receptive language and had more speech errors than children without NSCL/P. The magnitude of average effect sizes varied by subconstruct, and all were statistically significant (see Table 6). The study data and effect size data used in this study are available from the first author's ResearchGate profile.

**Table 6.** Intercept-only robust variance estimation model results and heterogeneity statistics by construct and subconstruct.

Subconstruct	*n*	*k*	ES_g_	*SE*	*df*	*p*	95% CI	tau^2^	*I* ^2^	*Q*
Speech										
Consonant inventory	26	9	−1.24	0.33	7.92	.01	[−2.01, −0.47]	1.01	85.87	56.62
Speech accuracy measures	44	12	−1.13	0.28	11	.01	[−1.74, −0.52]	1.32	90.19	112.13
Speech error measures	79	7	0.93	0.33	5.99	.03	[0.13, 1.74]	1.29	88.96	54.35
Language										
Expressive language	67	20	−0.57	0.07	17.1	< .001	[−0.72, −0.42]	0.08	49.35	37.51
Receptive language	21	13	−0.59	0.11	11.2	< .001	[−0.83, −0.35]	0.12	63.34	32.73

*Note.* Intercept-only robust variance estimation models were run to estimate the average effect size for each subconstruct. Nine separate models were run. For space concerns and comparison, all models are reported in one table. *n* = number of effect sizes; *k* = number of studies; ES_g_ = standardized mean difference effect size; *SE* = standard error; *df* = degrees of freedom; CI = confidence interval; tau^2^ = between-studies variance; *I* ^2^ = proportion of true between study variance; *Q* = measure of heterogeneity.

#### Consonant Inventory

The mean age of the samples was 21.20 months (*SD* = 5.29 months, range: 12–30 months). Effect sizes (*g*) for measures of consonant inventory ranged from −3.51 to −0.07. The intercept-only RVE model for consonant inventory revealed an average effect size of −1.24 (see Table 6), indicating that children with NSCL/P performed, on average, 1.24 *SD* units lower than children without NSCL/P on measures of consonant inventory.

Between-studies variance indicated that observed differences in effect sizes were likely greater than chance (see *I* ^2^ *,* τ^2^, and *Q* in Table 6). Thus, we conducted moderator analyses with age, cleft type, speech sample material (i.e., single word or connected speech), publication year, and study location to explore other sources of heterogeneity for consonant inventory measures. Table 8 displays single-predictor RVE metaregression models for consonant inventory measures. There were no moderators of effect size (see Table 7). Between-studies variances (τ^2^) across all models ranged from 0.81 (publication year) to 1.25 (speech sample material).

**Table 7.** Single-predictor metaregression models by construct and subconstruct.

Moderators	Speech subconstructs											
	Consonant inventory						Speech accuracy					
	*B*_0_	*B*	*SE*	*df*	*p*	95% CI	*B*_0_	*B*	*SE*	*df*	*p*	95% CI
Sample level												
*M*_age_	−0.89	−0.01	0.05	3.96	.79	[−0.17, 0.14]	−0.94	0	0.01	4.39	.68	[−0.03, 0.02]
Cleft type	−1.48	0.05	0.56	3.20	.93	[−1.66, 1.77]	**−1.80**	0.17	0.22	6.33	.47	[−0.37, 0.71]
Palatal repair age	−6.01	0.38	0.12	1.34	.14	[−0.45, 1.21]	−0.28	−0.07	0.27	1.16	.83	[−2.54, 2.40]
Palatal repair type	**−1.26**	0.14	0.39	6.94	.73	[−0.77, 1.04]	**−1.18**	0.19	0.55	6.51	.73	[−1.12, 1.52]
Study location	**−1.23**	−0.10	0.49	1.42	.86	[−3.35, 3.14]	**−1.30**	0.52	0.44	6	.28	[−0.55, 1.59]
Publication year	**−0.72**	−1.23	0.66	6.29	.11	[−2.82, 0.36]	**−0.95**	−0.23	0.39	3.33	.59	[−1.39, 0.93]
Measurement level												
Speech sample material	−0.21	−0.21	0.87	4.18	.82	[−2.57, 2.16]	**−1.82**	0.47	0.57	9.96	.42	[−0.79, 1.74]
	**Language subconstructs**											
	**Expressive language**						**Receptive language**					
**Moderators**	***B***_**0**_	***B***	***SE***	***df***	***p***	**95% CI**	***B***_**0**_	***B***	***SE***	***df***	***p***	**95% CI**
Sample level												
*M*_age_	**−0.85**	**0.01**	0.00	5.7	.01	[0.002, 0.01]	**−0.54**	0	0.00	4.94	.66	[−0.01, 0.00]
Cleft type	−0.64	0.02	0.07	7.64	.83	[−0.16, 0.19]	−0.73	0.03	0.11	2.49	.79	[−0.35, 0.41]
Study location	**−0.62**	0.16	0.13	8.21	.26	[−0.14, 0.45]	**−0.64**	0.18	0.15	3.45	.32	[−0.27, 0.63]
Publication year	**−0.61**	0.08	0.14	16.14	.57	[−0.22, 0.38]	**−0.74**	0.34	0.19	8.73	.12	[−0.11, 0.78]
Measurement level												
Assessment type	−0.16	**−0.29**	0.13	15.9	.05	[−0.57, −0.01]	—	—	—	—	—	—
Vocabulary vs. global testing	**−0.49**	−0.24	0.14	11.60	.11	[−0.54, 0.06]	**−0.49**	−0.44	0.33	2.89	.28	[−1.51, 0.63]

*Note.* All moderators were entered into single-predictor robust variance estimation models. Dashes indicate the variance were not included in the metaregression models. These variables were not applicable for the subconstruct. Bolded *B* values indicate statistically significant estimates at *p* < .05. Speech sample material was coded as single word versus connected speech. Assessment type was coded as parent report/naturalistic language sample versus standardized direct assessment. Language domain was coded as vocabulary versus omnibus language measure.

#### Speech Accuracy

The mean age of the samples was 44.20 months (*SD* = 24.90 months, range: 19–104 months). Effect sizes for measures of speech accuracy ranged from −6.67 to 1.06. The intercept-only RVE model for speech accuracy measures revealed an average effect size of −1.13 (see Table 6), indicating that children with NSCL/P performed, on average, 1.13 *SD* units lower than children without NSCL/P on measures of speech accuracy.

Between-studies variance indicated that observed differences in effect sizes were likely due to more than chance (see τ^2^ in Table 6). Thus, we conducted moderator analyses with age, cleft type, speech sample material (i.e., single word or connected speech), palatal repair age, palatal repair type (one-stage vs. two-stage repair), publication year, and study location to explore other sources of heterogeneity for speech accuracy measures. None of the moderators was significant (see Table 7). Between-studies variances (τ^2^) across all models ranged from 1.32 (speech sample material) to 2.45 (palatal repair age), indicating that these moderators did not explain the between-studies differences.

#### Speech Errors

The mean age of the samples was 55.10 months (*SD* = 7.59 months, range: 26–60 months). Effect sizes for speech errors ranged from −0.81 to 4.99. The intercept-only RVE model for speech errors revealed an average effect size of 0.93, indicating that children with NSCL/P produced, on average, 0.93 *SD* units more speech errors than children without NSCL/P. Although between-studies variance suggested that variability in effect sizes was likely more than chance (see τ^2^ in Table 6), there was insufficient power to conduct metaregressions for speech errors.

#### Expressive Language

This sample had a mean age of 43.79 months (*SD* = 23.90, range: 16.90–104 months). Effect sizes for expressive language ranged from −2.88 to 0.11. The intercept-only RVE model for expressive language revealed an average effect size of −0.57, indicating that, on average, children with NSCL/P performed 0.57 *SD* units lower than children without NSCL/P on measures of expressive language.

Between-studies variance was moderate (see *I* ^2^ in Table 7) but did not differ greatly between studies (see τ^2^ in Table 6). Thus, we conducted metaregressions for age, cleft type, language domain (vocabulary test vs. omnibus language test), assessment type (standardized vs. observational), study location, and publication year (see Table 7). Age was a statistically significant moderator for expressive language, indicating that older samples had smaller effect sizes. Specifically, a 1–*SD* unit change in age resulted in a 0.01 change in average effect size; thus, children with NSCL/P demonstrated more similar levels of performance on measures of expressive language compared to their peers as age increased. Assessment type was a statistically significant moderator for expressive language, indicating that effect sizes from observational and parent reports resulted in larger observed differences between children with and without NSCL/P than effect sizes calculated from standardized norm-referenced assessment data. Between-studies variance ranged from 0.05 (age) to 0.09 (cleft type and language domain).

#### Receptive Language

The mean age of the sample was 48.10 months (*SD* = 30.70, range: 16.890–104 months). Effect sizes ranged from −1.56 to 0.38 for measures of receptive language. The intercept-only RVE model revealed an average effect size of −0.59, indicating that, on average, children with NSCL/P performed 0.59 *SD* units lower than children without NSCL/P on measures of receptive language.

Between-studies variance indicated that differences in observed effect sizes were likely due to more than chance (see τ^2^ in Table 6). Thus, we conducted a moderator analysis examining age, cleft type, language domain (vocabulary vs. omnibus), study location, and publication year. None of the moderator metaregressions was significant (see Table 7). Between-studies variance ranged from 0.07 (language domain) to 0.14 (age); however, the true between-studies variance was small from the outset.

### Publication Bias

**Figure 2.**
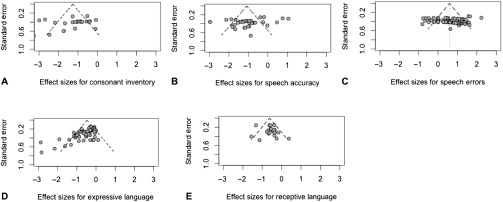
Funnel plots for (A) consonant inventory, (B) speech accuracy, (C) speech error, (D) expressive language, and (E) receptive language effect sizes.

To determine if the publication process biased our results (Chow & Ekholm, 2018), we examined publication bias by visually analyzing of a funnel plot where studies' effect sizes and standard errors were plotted, and Egger's regression and trim-and-fill analyses were run on appropriately powered samples. The funnel plots are presented in Figure 2. There was asymmetry observed for the speech accuracy and expressive language samples. Visual analysis for the speech accuracy sample revealed that there was little variability in the standard errors but that six effect sizes were outside the expected range in both directions; whereas, for the expressive language sample, the funnel plots indicated that effect sizes (*n* = 6) based on smaller sample sizes were larger. Table 8 summarizes the Egger's regression tests and trim-and-fill analyses. Based on the Egger's regression and trim-and-fill analyses, there was no evidence for publication bias for consonant inventory, speech error, or receptive language samples; however, there was moderate evidence of publication bias for speech accuracy and expressive language samples. For speech accuracy, the bias may be the result of small sample sizes, as most of the studies had an NSCL/P sample size of less than 20. The results for expressive language, however, are most likely due to missing literature, as the trim-and-fill analysis indicated that there were 17 studies missing, all with positive effect sizes.

**Table 8.** Summary of Egger's regression and trim-and-fill publication bias tests by subconstruct.

Sample	Egger's regression						Trim and fill		
	*t*	*df*	*p*	Bias	*SE* bias	Slope	Trimmed	Filled	Adj. standardized mean difference
Consonant inventory	−1.13	24	.2708	−2.32	2.06	−0.37	0	0	−1.34
Speech accuracy	−2.92	42	.0056	−6.31	2.16	1.16	0	0	−1.23
Speech error	0.94	76	.3489	1.69	1.79	−0.05	0	2	0.61
Expressive language	−642	65	< .0001	−2.93	0.46	0.28	0	17	−0.38
Receptive language	1.03	19	.3168	0.92	0.89	−0.84	0	5	−0.67

*Note. SE* = standard error; Adj. = Adjusted.

## Discussion

This meta-analysis investigated the performance of young children with NSCL/P on measures of speech articulation and language functioning and examined sample- and assessment-level moderators. Primary findings indicated that young children with NSCL/P performed significantly below their peers on measures of consonant inventory, speech accuracy, expressive language, and receptive language. Young children with NSCL/P had more speech errors than their peers. There was substantial variance across studies, most of which could not be explained by sample- or assessment-level moderators. Age and measurement type did not explain between-studies variance in expressive language skills.

### Deficits in Speech Articulation

Children with NSCL/P performed, on average, below their peers on all measures of speech articulation. The magnitude of differences varied across subconstructs, suggesting these three speech articulation measurement classes (e.g., speech accuracy, consonant inventory, and speech errors) captured unique information regarding differences in speech development. The largest discrepancy between children with NSCL/P and their peers was in consonant inventory, which is typically used to index development during the infant and toddler years when children are acquiring new consonants. Additionally, children with NSCL/P performed worse on measures of speech accuracy, which is consistent with previous literature (Klintö et al., 2014; Klintö, Salameh, & Lohmander, 2016; Lohmander & Persson, 2008). Finally, children with NSCL/P also demonstrated significantly more speech errors than their peers. One reason for the difference among the results for consonant inventory, speech accuracy, and speech error usage might be the age at time of assessment. Although all effect sizes were obtained from samples that were post-primary or first-stage palatal repair, there were substantial differences in the mean ages and age ranges for the different speech measure classes. The studies that reported consonant inventories had a limited age range (between 12 and 27 months); studies reporting speech accuracy and speech errors had wider age ranges and older mean ages (between 19.05 and 104 months).

None of our sample- or assessment-level moderators explained a significant amount of between-studies variance. This unexplained variance suggests that there is a considerable range in speech acquisition in individual children with NSCL/P. Some children progress rapidly in their speech development following palate repair, while others demonstrate greater variability in early consonant acquisition (Jones et al., 2003). Age was not a significant moderator for any of the speech measures despite the differences in age ranges for the data sets. On average, children with NSCL/P continued to have delayed speech development following palate repair. Speech accuracy in children with NSCL/P remained below the level achieved by children without clefts, and children with NSCL/P continued to present with speech errors across ages. This finding is consistent with results from the Americleft project, which reported that disordered speech skills persisted throughout early childhood and into school entry; 68% of 5- and 6-year-old children in the Americleft sample had below-average articulation skills for their age (Chapman et al., 2017). It is possible that other factors, such as hearing status or velopharyngeal function status, would have explained the between-studies variance. These factors were not consistently reported in the literature, and the unexplained variance in outcomes remains an area for future investigation.

### Lower Language Functioning and Moderating Factors

Children with NSCL/P had poorer expressive and receptive language skills compared to their peers. The magnitude of these effect sizes varied based on several factors: language modality assessed, age at time of assessment, and assessment type used. These findings are consistent with previous studies indicating that children with NSCL/P are delayed in language development (Broen, Devers, Doyle, Prouty, & Moller, 1998; Chapman, 2004; Hardin-Jones & Chapman, 2014; Morris & Ozanne, 2003; Nakajima, Mitsudome, & Yosikawa, 2001; Pamplona, Ysunza, González, Ramírez, & Patiño, 2000; Richman, 1980; Scherer, Boyce, et al., 2013; Scherer, Oravkinova, & McBee, 2013; Speltz et al., 2000; Young et al., 2010). Children with NSCL/P, on average, had expressive language scores that were half a standard deviation below their peers; however, age and assessment type moderated the effect sizes for expressive language. For age, effect sizes decreased as children with NSCL/P got older. The estimated average effect size was −0.62 for 36 months, −0.46 for 60 months, and −0.23 for 96 months. Similar decreases in effect sizes with age were evident across all language measures. Additionally, effect sizes obtained from parent reports or naturalistic observational samples were larger than those obtained from standardized direct assessments (the average effect size was −0.452 for standardized measures and −0.742 for parent reports or naturalistic observational samples). However, a complicating factor in interpreting these findings is that age and assessment type likely interact. There was a significant difference between the mean age for effect sizes drawn from parent report and naturalistic observational measures versus standardized direct assessments. Effect sizes for parent report and naturalistic language samples came from a younger population, which is most likely the result of the need to rely on parent reports and language samples because of limited language skills. For older children, researchers use standardized direct assessments, which measure language competence but do not provide information about functional language use in daily life, as may be indicated by parent report or observational language samples.

It is also possible that children with NSCL/P with typical cognitive skills, on average, have typical language skills compared to same-age peers (as measured by standardized norm-referenced tests), but their language use is different from that of their same-age, typically developing peers. This difference in functional use of language could be captured by observational measures (e.g., naturalistic, play-based language samples). For example, Frey et al. (2018) observed no differences between toddlers with and without NSCL/P on standardized norm-referenced assessments but significant differences on use of language in play-based contexts with familiar and unfamiliar communication partners. In this study, young children with NSCL/P spoke at lower rates and used less complex syntax compared to their age-matched peers but had similar language skills as measured by standardized, norm-referenced tests. Therefore, the meta-analysis finding that expressive language differences decreased with age might be an accurate indication of the overall expressive language competence of children with NSCL/P between the ages of 3 and 8 years but may not be not an accurate indicator of their use of language at home, in school, with peers, and/or with adults. Understanding how children with NSCL/P perform on a measure of functional language use (i.e., parent reports, naturalistic language samples) is important for developing individualized treatment goals and improving children's overall quality of living in their natural environments. Thus, a multimethod assessment approach is recommended to examine language skills of young children with NSCL/P. A combination of naturalistic and observational measures with standardized assessment measures would address evaluation of both language skill and language use (Frey et al., 2018).

The current meta-analysis indicated that children with NSCL/P have language vulnerabilities in the receptive modality compared to their peers. Receptive language skills did not improve as children with NSCL/P aged. When examining the mean standard score for receptive language across studies, most scores were within the normal range (i.e., did not exceed −1 *SD*s) even though children with NSCL/P differed from children without NSCL/P. It is clear from these results that children with NSCL/P have moderate but persistent delays in receptive language. While children who have global standardized language test scores within the normal range may not qualify for speech-language therapy in school settings, language skills in the borderline range of functioning are expected to affect children's academic functioning and peer relationships. Together, these findings for expressive and receptive language support a large body of research indicating persistent problems in language-related skills among children with NSCL/P (Hardin-Jones & Chapman, 2014; Morgan et al., 2017; Richman, 1980; Scherer, Boyce, et al., 2013; Watkins et al., 2018; Young et al., 2010). The examination of findings by age, assessment type, and language modality also provide some insight into the inconsistencies from past research findings.

### Publication Bias

Findings from the assessments of publication bias results did not raise any major concerns around the potential impact of the publication process on the analytic sample. Although, we had some evidence of possible bias, mainly for speech accuracy and expressive language, this evidence suggested only moderate bias. Analyses of these areas resulted in significant Egger's regression tests, suggesting that the current samples of data for speech accuracy and expressive language may contain smaller samples of studies. Furthermore, the trim-and-fill results suggest some imbalance in expressive language effect sizes relative to the mean. The values that were then filled represent smaller effect sizes, which align with performance closer to the typical range for children with NSCL/P. We interpret this potential bias with caution because we do not expect to find additional samples of children with NSCL/P who perform at typical levels for expressive language, and the funnel plot and Egger's regression tests for speech errors did not point to such bias. Finally, given this study is a meta-analysis of the average performance on measures and not of intervention effects, it is less likely that significant results from intervention studies influenced the current meta-analytic sample (Chow & Ekholm, 2018). Taken together, we conservatively estimate only a small to moderate influence of the publication process on our overall interpretation of our results.

### Study Quality—Potential Source of Bias

Study quality may be a potential source of bias in any meta-analysis. Meta-analyses of intervention studies typically include an assessment of study quality using a checklist from Cochrane guidelines or the Scottish Intercollegiate Guidelines Network. Such guidelines were developed for evaluating source of bias introduced as a function of experimental procedures in medical, drug, and clinical intervention studies. Sources of bias are evaluated because they may affect study quality and outcomes and include assessment of sequence generation, allocation concealment, blinding procedures, incomplete data reporting, and selective outcome reporting. These criteria are not applicable to descriptive and observational population-based studies included in this meta-analysis, because these studies did not include experimental procedures or manipulations in the study designs. Thus, these guidelines could not be not applied appropriately in the current analysis. Researchers are still working toward a conceptually similar study quality checklist for descriptive and observational studies (Page, Mckenzie, & Higgins, 2018; Sanderson, Tatt, & Higgins, 2007).

There are two aspects of studies that might have influenced our meta-analysis outcomes: incomplete data and transcription reliability. Studies in the current meta-analysis may have reported incomplete data for study participants due to a number of factors, including child refusal to participate, attrition, and otherwise missing data. A commonly accepted practice in the field is to use only complete data or when data are missing, impute values, and report the imputation procedures. In our corpus, the common practice was to use complete data only (25), use complete data per measure, and report test specific sample size (1) or not report if there was incomplete data (25). To address potential incomplete data, we used all available measures in a study with the reported sample size. Therefore, we feel confident that incomplete data had little to no effect on our results. Study quality could also be affected by whether or not researchers completed reliability assessments on speech or language transcription used to generate outcome measures. Transcription reliability is important for speech and language analyses, especially when using spontaneous speech and language sampling measures. In our corpus, 80% of speech studies and 91% of language studies reported interrater/intertranscriber reliability was assessed. Of the studies that reported reliability, 18 had reliability statistics over 80%, one reported no statistics, and one had an average reliability of 66%. Therefore, the effect of studies that did not report interrater/intertranscriber reliability is minimal and likely would not affect the average effect size estimates significantly.

### Limitations

There are several limitations to this study. First, some findings should be regarded as preliminary due to the small sample size for some analyses, as sample size varied for individual speech and language constructs (see *k* in Table 6) and for moderator analyses. For example, only seven studies reported speech error measures that could be included in our study, which resulted in a small degrees of freedom estimate. Additionally, some studies reported insufficient data for inclusion in our moderator analyses. Therefore, interpretation of our overall findings may be limited because each analysis used a subset of studies that included eligible data. Second, there was unexplained variance in the effect sizes across studies and outcomes, which, in some cases, was explored through moderator analyses. The moderator analyses did not always resolve the unexplained variance, which may indicate that between-studies differences arose from other sources, such as hearing status, socioeconomic status, or other less frequently reported characteristics important for speech and language development in children with NSCL/P. Third, the exploratory single-predictor metaregressions did not control for interaction between moderators. We did not have the power to conduct multiple predictor metaregressions to test for interactions between moderators, although there is evidence that some of them may have interacted (e.g., age and assessment method for expressive language). Despite these limitations, this meta-analysis extended the results from previous research by quantifying the differences in speech production and language development during early childhood and by providing insight into potential moderators of skill development.

### Implications for Practice

Based on the findings from this meta-analysis, multiple assessment measures should be used to characterize speech and language skill development in young children with NSCL/P. Different measurement tools yield unique information about speech and language functioning of young children with NSCL/P relative to their peers. Children with NSCL/P presented as more comparable to their peers for expressive language skills when direct standardized language assessments were used versus parent report or observational naturalistic measures. The average effect size for differences in expressive and receptive language were at the “subclinical” level, indicating that young children with NSCL/P would be unlikely to qualify for speech-language therapy services during the early school–age years, if this determination is made solely on the basis of standardized test scores. Speech articulation was found to be delayed across measures of consonant inventory, speech accuracy, and number of speech errors across the age range of children included in this meta-analysis. The reported measures provided a comprehensive assessment of children's speech development, and this range of measures should be used to assess children during early childhood. For children with NSCL/P, monitoring speech error usage (developmental errors and use of compensatory errors) is an important component of a speech assessment. Children with NSCL/P may show use of compensatory errors and/or phonological errors, and these error types have different implications for intervention.

### Implications for Future Research

In future studies, descriptions of sample characteristics should include age at palate repair, velopharyngeal function status at time of speech assessment, current hearing status and hearing history, history of the provision of speech and language services, socioeconomic status, and a description of child language environment. In terms of assessment characteristics, it is important to report speech sample materials used during assessment and to include consonant inventory, speech accuracy, and speech error usage. For language, we recommend that researchers include parent reports of language development and naturalistic language samples in addition to the standardized direct assessments of language. This meta-analysis found differences in effect sizes when comparing standardized direct assessments to parent report measures and language samples. Few studies included all three of these language measure types. The observed differences between measurement types might account for reported differences in language development in the literature. Parent reports, language samples, and standardized direct assessments of language each provides distinctive information about language that, when used in combination, will advance understanding of language development in children with NSCL/P. While this meta-analysis provides information on speech and language functioning at single time points during early development, a synthesis of the longitudinal work is needed to examine the trajectory of speech and language development in this clinical population with risk of delays in these domains. The finding that persistent deficits in speech articulation and receptive language were not completely not moderated by age in the current analysis highlights this critical need for longitudinal studies and syntheses of extant longitudinal research.

## Conclusions

Results of this meta-analysis suggest that children with NSCL/P have impairments in both speech and language development characterized by reduced consonant inventory and speech accuracy, more speech errors, and deficits in expressive and receptive language functioning. Speech skills and receptive language functioning did not improve with age, indicating that children with NSCL/P consistently demonstrate lower speech and receptive language skills than their peers throughout early childhood. Age and assessment type moderated expressive language functioning in this sample, highlighting the importance of considering timing and method of assessing expressive language skills. This meta-analysis provides important information for future research studies and current clinical practice. The single most important finding from this study is that children with NSCL/P have persistent speech and language deficits during early childhood. Furthermore, the timing and methods used to assess speech and language skills may affect assessment outcomes.

## Supplementary Material

10.1044/2019_JSLHR-19-00162SMS1 Supplemental Material 1. PRISMA 2009 Checklist.

10.1044/2019_JSLHR-19-00162SMS2 Supplemental Material 2. Code for meta-analysis.

10.1044/2019_JSLHR-19-00162SMS3 Supplemental Material 3. List of citations and study ID number for included studies.
