# Supplementary material for: Early Speech and Language Development in Children With Nonsyndromic Cleft Lip and/or Palate: A Meta-Analysis
Source: J Speech Lang Hear Res. 2019 Dec 13;63(1):14–31. doi: 10.1044/2019_JSLHR-19-00162 (PMC7213476; doi:10.1044/2019_JSLHR-19-00162)
Supplement: Supplemental Material 2. [file JSLHR-63-14-s002.pdf]

Supplemental Material S2. Code for Meta-Analysis.

Load libraries

```
suppressMessages({library(dplyr)
library(meta)
library(readxl)
library(robumeta)
library(grid)
library(irr)})
```

Load data

```
cleft_meta_study <- read_delim("~/SuppD_studydata.txt", "\t", escape_double =
FALSE, trim_ws = TRUE)

cleft_meta <- read_delim("~/SuppE_studydata.txt","\t", escape_double = FALSE,
trim_ws = TRUE)
```

Prepare data

The following several code chunks prepare the data for analysis.

Adding study label

This section of code creates a study label based on the first author's last name and the publication year (e.g., Broen 1998).

```
# create study label variable
cleft_meta_study$study_lab <- paste(cleft_meta_study$author, cleft_meta_study
$year)

# create df with just study_id and study_lable
study_label <- cleft_meta_study %>%
  select(study_id, study_lab)
```

This code chunk appends the study label to the study data dataframe.

```
cleft_meta$study_id <- as.character(cleft_meta$study_id)

cleft_meta$name <- as.factor(cleft_meta$name)
```

Create standard error terms

This code chunk creates the standard error terms for the effect sizes.

```
cleft_meta <- cleft_meta %>%
  mutate(eff_se = sqrt(var))
```

Transform d --> g

This code chunk transforms Cohen's d effect sizes to Hedge's g.

```
d_to_g <- function(eff_d, n1, n2){
  eff_d * (1 - (3 / (4 * (n1 + n2) - 9)))
}

cleft_meta <- cleft_meta %>%
  mutate(eff_g = d_to_g(eff_size, cleft_n, compare_n))
```

Subset data

This code chunk subsets the effect size data into each subconstruct for later analysis.

```
con <- cleft_meta %>%
  filter(sub_construct == 11)

acc <- cleft_meta %>%
  filter(sub_construct == 12)

error <- cleft_meta %>%
  filter(sub_construct == 13) %>%
  filter(!is.na(eff_size))

recep_d <- cleft_meta %>%
  filter(sub_construct == 21)

express_d <- cleft_meta %>%
  filter(sub_construct == 22) %>%
  filter(study_id != 11)
```

Study data

The following code chunks summarize the demographic information about the total sample, the speech studies only, and the language studies only. These data were used to create Table 1.

Total sample

```
incl_s <- unique(cleft_meta$study_id)

cleft_meta_study %>%
  filter(study_id %in% incl_s) %>%
  mutate(total_n = cleft_n + comp_n) %>%
  summarise(n_studies = n(),
            reli_n = sum(reliability == 1, na.rm = T),
            synd_n = sum(syndrome_exclude == 1, na.rm = T),
            group_n = sum(study_design == 2 | study_design == 4),
            long_n = sum(study_design == 5),
            int_n = sum(study_design == 1 | study_design == 3),
            tot_n = sum(total_n, na.rm=T),
            mean_cleft_age = mean(cleft_age_m, na.rm=T),
            sd_cleft_age = sd(cleft_age_m, na.rm = T),
            mean_compare_age = mean(comp_age_m, na.rm=T),
            sd_compare_age = sd(comp_age_m, na.rm = T),
            tot_cleft_n = sum(cleft_n, na.rm=T),
            tot_compare_n = sum(comp_n, na.rm=T),
            mean_pal = mean(pal_repair_age, na.rm = T),
            usa = sum(international == 0),
            loc_other = sum(international == 1 | international == 9),
            pub_06 = sum(year >= 2006),
            pub_05 = sum(year < 2006),
            pal_sd = sd(pal_repair_age, na.rm = T),
            per_m_w = mean(per_male_whole, na.rm = T),
            per_sd_w = sd(per_male_whole, na.rm = T),
            per_m_c = mean(per_male_cleft, na.rm = T),
            per_sd_c = sd(per_male_cleft, na.rm = T))
```

Speech studies

```
speech_s <- cleft_meta %>%
  filter(construct == 1)

speech_s <- unique(speech_s$study_id)

cleft_meta_study %>%
  filter(study_id %in% speech_s) %>%
  mutate(total_n = cleft_n + comp_n) %>%
  summarise(n_studies = n(),
            reli_n = sum(reliability == 1, na.rm = T),
            synd_n = sum(syndrome_exclude == 1, na.rm = T),
            group_n = sum(study_design == 2 | study_design == 4),
            long_n = sum(study_design == 5),
            int_n = sum(study_design == 1 | study_design == 3),
            tot_n = sum(total_n, na.rm=T),
            mean_cleft_age = mean(cleft_age_m, na.rm=T),
            sd_cleft_age = sd(cleft_age_m, na.rm = T),
            mean_compare_age = mean(comp_age_m, na.rm=T),
```

```
            sd_compare_age = sd(comp_age_m, na.rm = T),
            tot_cleft_n = sum(cleft_n, na.rm=T),
            tot_compare_n = sum(comp_n, na.rm=T),
            mean_pal = mean(pal_repair_age, na.rm = T),
            usa = sum(international == 0),
            loc_other = sum(international == 1 | international == 9),
            pub_06 = sum(year >= 2006),
            pub_05 = sum(year < 2006),
            pal_sd = sd(pal_repair_age, na.rm = T),
            per_m_w = mean(per_male_whole, na.rm = T),
            per_sd_w = sd(per_male_whole, na.rm = T),
            per_m_c = mean(per_male_cleft, na.rm = T),
            per_sd_c = sd(per_male_cleft, na.rm = T))
```

Language

```
lang_s <- cleft_meta %>%
  filter(construct == 2)

lang_s <- unique(lang_s$study_id)

cleft_meta_study %>%
  filter(study_id %in% lang_s) %>%
  mutate(total_n = cleft_n + comp_n) %>%
  summarise(n_studies = n(),
            reli_n = sum(reliability == 1, na.rm = T),
            synd_n = sum(syndrome_exclude == 1, na.rm = T),
            group_n = sum(study_design == 2 | study_design == 4),
            long_n = sum(study_design == 5),
            int_n = sum(study_design == 1 | study_design == 3),
            tot_n = sum(total_n, na.rm=T),
            mean_cleft_age = mean(cleft_age_m, na.rm=T),
            sd_cleft_age = sd(cleft_age_m, na.rm = T),
            mean_compare_age = mean(comp_age_m, na.rm=T),
            sd_compare_age = sd(comp_age_m, na.rm = T),
            tot_cleft_n = sum(cleft_n, na.rm=T),
            tot_compare_n = sum(comp_n, na.rm=T),
            mean_pal = mean(pal_repair_age, na.rm = T),
            usa = sum(international == 0),
            loc_other = sum(international == 1 | international == 9),
            pub_06 = sum(year >= 2006),
            pub_05 = sum(year < 2006),
            pal_sd = sd(pal_repair_age, na.rm = T),
            per_m_w = mean(per_male_whole, na.rm = T),
            per_sd_w = sd(per_male_whole, na.rm = T),
            per_m_c = mean(per_male_cleft, na.rm = T),
            per_sd_c = sd(per_male_cleft, na.rm = T))
```

Meta results

The following code chunks are the code used to preform the intercept-only random effect models and the single-predictor regression analyses. Each subconstruct is analyzed in one code chunk. The output for these codes are the results seen in Tables 6 and 7.

Consonant inventory

```
con_m <- robu(eff_g ~ 1, con, studynum = study_id, var.eff.size = var, rho =
0.8, small = TRUE)

con_m

con_regage <- robu(eff_g ~ age, con, studynum = study_id, var.eff.size = var,
rho = 0.8, small = TRUE)

con_regage

con_regtype <- robu(eff_g ~ sample_context, con, studynum = study_id, var.eff
.size = var, rho = 0.8, small = TRUE)

con_regtype

con_regcleft <- robu(eff_g ~ cleft_type, con, studynum = study_id, var.eff.si
ze = var, rho = 0.8, small = TRUE)

con_regcleft

con_loc <- robu(eff_g ~ location, con, studynum = study_id, var.eff.size = va
r, rho = 0.8, small = TRUE)

con_loc

con_year <- robu(eff_g ~ year, con, studynum = study_id, var.eff.size = var,
rho = 0.8, small = TRUE)

con_year
```

Speech accuracy

```
acc_m <- robu(eff_g ~ 1, acc, studynum = study_id, var.eff.size = var, rho =
0.8, small = TRUE)

acc_m

acc_regage <- robu(eff_g ~ age, acc, studynum = study_id, var.eff.size = var,
rho = 0.8, small = TRUE)

acc_regage
```

```
acc_regtype <- robu(eff_g ~ sample_context, acc, studynum = study_id, var.eff
.size = var, rho = 0.8, small = TRUE)

acc_regtype

acc_regcleft <- robu(eff_g ~ cleft_type, acc, studynum = study_id, var.eff.si
ze = var, rho = 0.8, small = TRUE)

acc_regcleft

acc_loc <- robu(eff_g ~ location, acc, studynum = study_id, var.eff.size = va
r, rho = 0.8, small = TRUE)

acc_loc

acc_year <- robu(eff_g ~ year, acc, studynum = study_id, var.eff.size = var,
rho = 0.8, small = TRUE)

acc_year
```

Speech Errors

```
error_m <- robu(eff_g ~ 1, error, studynum = study_id, var.eff.size = var, rh
o = 0.8, small = TRUE)

error_m

error_regage <- robu(eff_g ~ age, error, studynum = study_id, var.eff.size =
var, rho = 0.8, small = TRUE)

error_regage

error_regtype <- robu(eff_g ~ sample_context, error, studynum = study_id, var
.eff.size = var, rho = 0.8, small = TRUE)

error_regtype

error_regcleft <- robu(eff_g ~ cleft_type, error, studynum = study_id, var.ef
f.size = var, rho = 0.8, small = TRUE)

error_regcleft

error_loc <- robu(eff_g ~ location, error, studynum = study_id, var.eff.size
= var, rho = 0.8, small = TRUE)

error_loc

error_year <- robu(eff_g ~ year, error, studynum = study_id, var.eff.size = v
```

```
ar, rho = 0.8, small = TRUE)

error_year
```

Receptive language

```
recep_m <- robu(eff_g ~ 1, recep_d, studynum = study_id, var.eff.size = var,
rho = 0.8, small = TRUE)

recep_m

recep_regression <- robu(eff_g ~ age, recep_d, studynum = study_id, var.eff.s
ize = var, rho = 0.8, small = TRUE)

recep_regression

recep_regvocab <- robu(eff_g ~ sample_context, recep_d, studynum = study_id,
var.eff.size = var, rho = 0.8, small = TRUE)

recep_regvocab

recep_regcleft <- robu(eff_g ~ cleft_type, recep_d, studynum = study_id, var.
eff.size = var, rho = 0.8, small = TRUE)

recep_regcleft

recep_loc <- robu(eff_g ~ location, recep_d, studynum = study_id, var.eff.siz
e = var, rho = 0.8, small = TRUE)

recep_loc

recep_year <- robu(eff_g ~ year, recep_d, studynum = study_id, var.eff.size =
var, rho = 0.8, small = TRUE)

recep_year
```

Expressive language

```
express_m <- robu(eff_g ~ 1, express_d, studynum = study_id, var.eff.size = v
ar, rho = 0.8, small = TRUE)

express_m

express_rage <- robu(eff_g ~ age, express_d, studynum = study_id, var.eff.siz
e = var, rho = 0.8, small = TRUE)

express_rage

express_rvocab <- robu(eff_g ~ sample_context, express_d, studynum = study_id
, var.eff.size = var, rho = 0.8, small = TRUE)
```

```
express_rvocab

express_rmeasure <- robu(eff_g ~ measure_type, express_d, studynum = study_id
, var.eff.size = var, rho = 0.8, small = TRUE)

express_rmeasure

express_rtype <- robu(eff_g ~ cleft_type, express_d, studynum = study_id, var
.eff.size = var, rho = 0.8, small = TRUE)

express_rtype

express_loc <- robu(eff_g ~ location, express_d, studynum = study_id, var.eff
.size = var, rho = 0.8, small = TRUE)

express_loc

express_year <- robu(eff_g ~ year, express_d, studynum = study_id, var.eff.si
ze = var, rho = 0.8, small = TRUE)

express_year
```

Publication bias

The following code chunks analyze the subconstructs for publication bias. These results are presenting in Table 8 and Figure 2.

Consonant Inventory

Egger test

```
metabias(con$eff_g, con$eff_se, method.bias = "linreg", plotit = TRUE)
```

Funnel plot

```
con_fun <- funnel(con$eff_g, con$eff_se, xlab = "Effect Sizes for Consonant I
nventory", ylab = "Standard Error", sm = "SMD", axes = TRUE, col.random = "re
d", yaxis = "se", level = 0.95, xlim = c(-3, 3), ylim = c(1, 0))
```

Trim and fill

```
trimfill(con$eff_g, con$eff_se, ma.fixed = FALSE, sm = "SMD", comb.random = T
RUE)
```

Speech accuracy

Egger test

```
metabias(acc$eff_g, acc$eff_se, method.bias = "linreg", plotit = TRUE)
```

Funnel plot

```
acc_fun <- funnel(acc$eff_g, acc$eff_se, xlab = "Effect Sizes for Speech Accu
racy", ylab = "Standard Error", sm = "SMD", axes = TRUE, col.random = "red",
yaxis = "se", level = 0.95, xlim = c(-3, 3), ylim = c(1, 0))
```

Trim and fill

```
trimfill(acc$eff_g, acc$eff_se, ma.fixed = FALSE, sm = "SMD", comb.random = T
RUE)
```

Speech error

Egger test

```
metabias(error$eff_g, error$eff_se, method.bias = "linreg", plotit = TRUE)
```

Funnel

```
error_fun <- funnel(error$eff_g, error$eff_se, comb.random = TRUE, xlim = c(-
3, 3), ylim = c(1, 0), level = 0.95, xlab = "Effect Sizes for Speech Errors",
ylab = "Standard Error", sm = "SMD", axes = TRUE)
```

Trim and fill

```
trimfill(error$eff_g, error$eff_se, sm = "SMD")
```

Expressive Language

Egger test

```
metabias(express_d$eff_g, express_d$eff_se, method.bias = "linreg", plotit =
TRUE)
```

Funnel

```
express_fun <- funnel(express_d$eff_g, express_d$eff_se, xlab = "Effect Sizes
for Expressive Language", ylab = "Standard Error", sm = "SMD", axes = TRUE, c
ol.random = "red", yaxis = "se", level = 0.95, xlim = c(-3, 3), ylim = c(1, 0
))
```

Trim and fill

```
trimfill(express_d$eff_g, express_d$eff_se, ma.fixed = FALSE, sm = "SMD", com
b.random = TRUE)
```

Receptive Language

Egger test

```
metabias(recep_d$eff_g, recep_d$eff_se, method.bias = "linreg", plotit = TRUE
)
```

Funnel

```
recep_fun <- funnel(recep_d$eff_g, recep_d$eff_se, xlab = "Effect Sizes for R
eceptive Language", ylab = "Standard Error", sm = "SMD", axes = TRUE, col.ran
dom = "red", yaxis = "se", level = 0.95, xlim = c(-3, 3), ylim = c(1, 0))
```

Trim and fill

```
trimfill(recep_d$eff_g, recep_d$eff_se, ma.fixed = FALSE, sm = "SMD", comb.ra
ndom = TRUE)
```
