# Supplementary material for: Early Speech and Language Development in Children With Nonsyndromic Cleft Lip and/or Palate: A Meta-Analysis
Source: J Speech Lang Hear Res. 2019 Dec 13;63(1):14–31. doi: 10.1044/2019_JSLHR-19-00162 (PMC7213476; doi:10.1044/2019_JSLHR-19-00162)
Supplement: Supplemental Material 3. [file JSLHR-63-14-s003.pdf]

Supplemental Material S3. List of citations and study ID number for included studies.

**Citation**	**Study ID**
Broen, P. A., Devers, M. C., Doyle, S. S., Prouty, J. M., & Moller, K. T. (1998). Acquisition of linguistic and cognitive skills by children with cleft palate. *Journal of Speech, Language, and Hearing Research*, *41*(3), 676–687. https://doi.org/10.1044/jslhr.4103.676	2
Chapman, K. L., Graham, K. T., Gooch, J., & Visconti, C. (1998). Conversational skills of preschool and school-age children with cleft lip and palate. *The Cleft Palate-Craniofacial Journal*, *35*, 503–516. https://doi.org/10.1597/1545-1569_1998_035_0503_csopas_2.3.co_2	3
Chapman, K. L., & Hardin-Jones, M. A. (1992). Phonetic and phonological skills of two-year-olds with cleft palate. *The Cleft Palate-Craniofacial Journal*, *29*(5), 435–443. https://doi.org/10.1597/1545-1569_1992_029_0435_papsot_2.3.co_2	73
Chapman, K. L., Hardin-Jones, M. A., & Halter, K. A. (2003). The relationship between early speech and later speech and language performance for children with cleft lip and palate. *Clinical Linguistics & Phonetics*, *17*(3), 173–197. https://doi.org/10.1080/0269920021000047864	4
Chapman, K. L. (2011). The relationship between early reading skills and speech and language performance in young children with cleft lip and palate. *The Cleft Palate-Craniofacial Journal*, *48*(3), 301–311. https://doi.org/10.1597/08-213	5
Collett, B. R., Leroux, B., & Speltz, M. L. (2010). Language and early reading among children with orofacial clefts. *The Cleft Palate-Craniofacial Journal*, *47*, 284–292. https://doi.org/10.1597/08-172.1	9
Estrem, T., & Broen, P. A. (1987). Early speech production of children with cleft palate. *Journal of Speech and Hearing Research*, *32*(1), 12–23. https://doi.org/10.1044/jshr.3201.12	56
Fox, D., Lynch, J., & Brookshire, B. (1978). Selected developmental factors of cleft palate children between two and thirty-three months of age. *The Cleft Palate Journal*. Retrieved from http://www.ncbi.nlm.nih.gov/pubmed/282024	55
Hardin-Jones, M. A., & Chapman, K. L. (2014). Early lexical characteristics of toddlers with cleft lip and palate. *The Cleft Palate-Craniofacial Journal*, *51*(6), 622–631. https://doi.org/10.1597/13-076	15
Hentges, F., Hill, J., Bishop, D. V. M., Goodacre, T., Moss, T., & Murray, L. (2011). The effect of cleft lip on cognitive development in school-aged children: A paradigm for examining sensitive period effects. *Journal of Child Psychology and Psychiatry and Allied Disciplines*, *52*(6), 704–712. https://doi.org/10.1111/j.1469-7610.2011.02375.x	16
Jones, C. E., Chapman, K. L., & Hardin-Jones, M. A. (2003). Speech development of children with cleft palate before and after palatal surgery. *The Cleft Palate-Craniofacial Journal*, *40*(1), 19–31. https://doi.org/10.1597/1545-1569_2003_040_0019_sdocwc_2.0.co_2	18

Jocelyn, L. J., Penko, M. A., & Rode, H. L. (1996). Cognition, communication, and hearing in young children with cleft lip and palate and in control children: A longitudinal study. *Pediatrics*, *97*(4), 529–534.	98
Klintö, K., Salameh, E.-K., & Lohmander, A. (2015). Verbal competence in narrative retelling in 5-year-olds with unilateral cleft lip and palate. *International Journal of Language and Communication Disorders*, *50*(1), 119–128. https://doi.org/10.1111/1460-6984.12127	19
Klintö, K., Salameh, E.-K., & Lohmander, A. (2016). Phonology in Swedish-speaking 5-year-olds born with unilateral cleft lip and palate and the relationship with consonant production at 3 years of age. *International Journal of Speech-Language Pathology*, *18*(2), 147–156. https://doi.org/10.3109/17549507.2015.1081287	20
Klintö, K., Salameh, E.-K., Svensson, H., & Lohmander, A. (2011). The impact of speech material on speech judgement in children with and without cleft palate. *International Journal of Language & Communication Disorders*, *46*(3), 348–360. https://doi.org/10.3109/13682822.2010.507615	57
Kummer, A. W., Lee, L., Stutz, L. S., Maroney, A., & Brandt, J. W. (2007). The prevalence of apraxia characteristics in patients with velocardiofacial syndrome as compared with other cleft populations. *The Cleft Palate-Craniofacial Journal*, *44*(2), 175–181. https://doi.org/10.1597/05-170.1	93
Lee, H.-C., Madison, C. L., & Jackson, R. (2014). Phonological competence in Mandarin Chinese-speaking children with cleft palate. *Asia Pacific Journal of Speech, Language and Hearing*, *12*(1), 13–26. https://doi.org/10.1179/jslh.2009.12.1.13	90
Lee, K. S. M., Young, S. E.-L., Liow, S. J. R., & Purcell, A. A. (2015). Spelling processes of children with nonsyndromic cleft lip and/or palate: A preliminary study. *The Cleft Palate-Craniofacial Journal*, *52*(1), 70–81. https://doi.org/10.1597/13-120	22
Lohmander, A., & Persson, C. (2008). A longitudinal study of speech production in Swedish children with unilateral cleft lip and palate and two-stage palatal repair. *The Cleft Palate-Craniofacial Journal*, *45*(1), 32–41. https://doi.org/10.1597/06-123.1	23
Luyten, A., Bettens, K., D'haeseleer, E., De Ley, S., Hodges, A., Galiwango, G., … Van Lierde, K. (2014). The impact of palatal repair before and after 6 months of age on speech characteristics. *International Journal of Pediatric Otorhinolaryngology*, *78*(5), 787–798. https://doi.org/10.1016/j.ijporl.2014.02.012	84
Luyten, A., Bettens, K., D'haeseleer, E., De Ley, S., Hodges, A., Galiwango, G., … Van Lierde, K. (2014). Impact of early synchronous lip and palatal repair on speech. *Folia Phoniatrica et Logopaedica*, *65*(6), 303–311. https://doi.org/10.1159/000362501	24
Nakajima, T., Mitsudome, A., & Yosikawa, A. (2001). Postoperative speech development based on cleft types in children with cleft palate. *Pediatrics International, 43*(6), 666–672. https://doi.org/10.1046/j.1442-200X.2001.01478.x	28
Philips, B. J., & Harrison, R. J. (1969). Language skills of preschool cleft palate	49

children. *The Cleft Palate Journal*. Retrieved from http://www.ncbi.nlm.nih.gov/pubmed/5253573	
Priester, G. H., & Goorhuis-Brouwer, S. M. (2008). Speech and language development in toddlers with and without cleft palate. *International Journal of Pediatric Otorhinolaryngology*, *72*(6), 801–806. https://doi.org/10.1016/j.ijporl.2008.02.004	91
Scherer, N. J., D'Antonio, L. L., & McGahey, H. (2008). Early intervention for speech impairment in children with cleft palate. *The Cleft Palate-Craniofacial Journal*, *45*(1), 18–31. https://doi.org/10.1597/06-085.1	37
Scherer, N. J., Williams, L., Stoel-Gammon, C., & Kaiser, A. P. (2012). Assessment of single-word production for children under three years of age: Comparison of children with and without cleft palate. *International Journal of Otolaryngology*, *2012*, Article ID 724214. https://doi.org/10.1155/2012/724214	38
Scherer, N. J., Oravkinova, Z., & McBee, M. T. (2013). Longitudinal comparison of early speech and language milestones in children with cleft palate: A comparison of US and Slovak children. *Clinical Linguistics & Phonetics*, *27*(6–7), 404–418. https://doi.org/10.3109/02699206.2013.769024	40
Scherer, N. J., Williams, A. L., & Proctor-Williams, K. (2008). Early and later vocalization skills in children with and without cleft palate. *International Journal of Pediatric Otorhinolaryngology*, *72*(6), 827–840. https://doi.org/10.1016/j.ijporl.2008.02.010	72
Scherer, N., & D'Antonio, L. L. (1995). Parent questionnaire for screening early language development in children with cleft palate. *The Cleft Palate-Craniofacial Journal*. https://doi.org/10.1597/1545-1569_1995_032_0007_pqfsel_2.3.co_2	51
Speltz, M. L., Endriga, M. C., Hill, S., Catherine, L., Jones, K., & Omnell, M. L. (2000). Brief report: Cognitive and psychomotor development of infants with orofacial clefts. *Journal of Pediatric Psychology*, *25*(3), 185–190. https://doi.org/10.1093/jpepsy/25.3.185	66
Snyder, L. E., & Scherer, N. J. (2004). The development of symbolic play and language in toddlers with cleft palate. *American Journal of Speech-Language Pathology*, *13*(1), 66–80. https://doi.org/10.1044/1058-0360(2004/008)	42
Willadsen, E., & Enemark, H. (2000). A comparative study of prespeech vocalizations in two groups of toddlers with cleft palate and a noncleft group. *The Cleft Palate-Craniofacial Journal*, *37*(2), 172–178. https://doi.org/10.1597/1545-1569_2000_037_0172_acsopv_2.3.co_2	59
